# Elements of Surgical Diagnosis

**Published:** 1885-01

**Authors:** 


**Elements of Surgical Diagnosis.** By A. PEARCE GOULD, M. S., M. D., London; F. R. C. S., England; Assistant Surgeon to the Middlesex Hospital, London, etc. Philadelphia: Henry C. Lea's Sons & Co. 1884.

This little work belongs to the series of manuals for students of medicine, and contains a condensed epitome of surgical diagnosis. For students who, in their earlier course, have but limited leisure for study, this compend offers many advantages. We commend the work to medical students, for whom it is written.

